# *Toxoplasma gondii* Proliferation Require Down-Regulation of Host Nox4 Expression via Activation of PI3 Kinase/Akt Signaling Pathway

**DOI:** 10.1371/journal.pone.0066306

**Published:** 2013-06-18

**Authors:** Wei Zhou, Juan-Hua Quan, Young-Ha Lee, Dae-Whan Shin, Guang-Ho Cha

**Affiliations:** 1 Department of Infection Biology, Chungnam National University School of Medicine, Daejeon, Korea; 2 Department of Gastroenterology, The Affiliated Hospital of Guangdong Medical College, Zhanjiang, Guangdong, China; University of Oklahoma Health Sciences Center, United States of America

## Abstract

*Toxoplasma gondii* results in ocular toxoplasmosis characterized by chorioretinitis with inflammation and necrosis of the neuroretina, pigment epithelium, and choroid. After invasion, *T. gondii* replicates in host cells before cell lysis, which releases the parasites to invade neighboring cells to repeat the life cycle and establish a chronic retinal infection. The mechanism by which *T. gondii* avoids innate immune defense, however, is unknown. Therefore, we determined whether PI3K/Akt signaling pathway activation by *T. gondii* is essential for subversion of host immunity and parasite proliferation. *T. gondii* infection or excretory/secretory protein (ESP) treatment of the human retinal pigment epithelium cell line ARPE-19 induced Akt phosphorylation, and PI3K inhibitors effectively reduced *T. gondii* proliferation in host cells. Furthermore, *T. gondii* reduced intracellular reactive oxygen species (ROS) while activating the PI3K/Akt signaling pathway. While searching for the main source of these ROS, we found that NADPH oxidase 4 (Nox4) was prominently expressed in ARPE-19 cells, and this expression was significantly reduced by *T. gondii* infection or ESP treatment along with decreased ROS levels. In addition, artificial reduction of host Nox4 levels with specific siRNA increased replication of intracellular *T. gondii* compared to controls. Interestingly, these *T. gondii*-induced effects were reversed by PI3K inhibitors, suggesting that activation of the PI3K/Akt signaling pathway is important for suppression of both Nox4 expression and ROS levels by *T. gondii* infection. These findings demonstrate that manipulation of the host PI3K/Akt signaling pathway and Nox4 gene expression is a novel mechanism involved in *T. gondii* survival and proliferation.

## Introduction

*Toxoplasma gondii* is one of the most widespread zoonotic pathogens in the world. The tachyzoite is a rapidly dividing haploid form of *T. gondii* that can infect a wide range of mammalian host cells, including immune and non-immune cells [1], [2]. Inside the host cell, parasites reside within a specialized parasitophorous vacuole (PV) that resists endosomal acidification and lysosomal fusion, and these parasites exhibit rapid intracellular replication, redistributes host intracellular organelles and cytoskeleton, and modulates host cell gene expression [3] in the PV. *T. gondii* is an obligate intracellular parasite that competes with host cells for metabolites, such as glucose, lipids, and amino acids, as well as nucleotides for its survival [4]. To win this fierce competition for survival, the parasite displays a highly sophisticated ability to distort host responses and their underlying signal transduction cascades. However, the cellular factors involved in its intracellular replication are not well defined.

Recently, several reports demonstrated that the host PI3K/Akt signaling pathway is stimulated by *T. gondii* infection [5]. PI3K is a ubiquitously expressed enzyme that is responsible for the regulation of various intracellular processes, such as insulin-dependent cell growth, membrane trafficking, and endosome fusion [6]. The serine/threonine protein kinase B (PKB)/Akt is one of the major downstream targets of PI3K and is a central player in growth regulation of cells [7]. Phosphorylation at Ser473 and Thr308 activates the kinase activity of Akt, which regulates multiple cellular processes that increase metabolism, growth, and synthetic processes and suppress apoptosis [8]. PI3K/Akt signaling plays an important role in *T. gondii* invasion of host cells because phosphatidylinositol (3, 4, 5)-trisphosphate (PIP3) rapidly accumulates in host cells in response to infective tachyzoites and more importantly, PI3K inhibitors partially reduce parasite entry [9], [10]. *Another study showed that* excretory/secretory proteins (ESP) from *T. gondii* play an important role in generating suitable conditions for parasite invasion into host cells [11].

Ocular toxoplasmosis is an inflammatory process that involves the interior of the eye and is caused by infection with *T. gondii*. This disease is the most common posterior uveitis in immunocompent individuals [12]. Although ocular toxoplasmosis usually consists of a self-limited retinochoroiditis, sight-threatening complications occur, and this disease is the foremost cause of unilateral vision loss in patients with uveitis [13]. The intensity of damage to the retina and choroid depends on the severity of infection and associated inflammatory reaction. In the most severe form of *T. gondii*-induced uveitis, degeneration of large segments of the outer retina and pigment epithelium are often observed [14].

The retinal pigment epithelium (RPE) forms a single-cell layer between the retinal photoreceptors and the vascular-rich choroids, thereby forming the blood-retinal barrier. RPE normally functions under relatively high oxygen tensions in postnatal life. Several animal models showed that *T. gondii* tachyzoites reach the retina via both choroidal and retinal circulation. Upon injection of tachyzoites into the suprachoroidal space in rabbits, outer retinal lesions and localized foci of retinal pigment epitheliosis were observed within 48 h [15]. This result suggests that tachyzoites penetrate the RPE-Bruchs membrane barrier from the choroid to the retina. Histopathological examination of toxoplasma retinochoroiditis patients showed the presence of free tachyzoites and cysts in the RPE and the retina [16]. These reports support the idea that RPE is one of the preferred infection sites for *T. gondii*, although whether the RPE is an ideal environment for *T. gondii* survival and proliferation is not known. Therefore, in the present study, we used a human RPE cell line (ARPE-19) as a model for ocular toxoplasmosis to study host defense in response to parasite invasion and intracellular replication.

Reactive oxygen species (ROS), which occurs as byproducts of metabolic reactions, normally participate in various cellular physiological events. ROS regulate certain essential signaling pathway, targeting Akt [17], NF-κB [18] and MAPK [19], etc. However, ROS are chemically reactive and therefore, can easily damage DNA, lipids, and proteins when present at high concentrations. Therefore, high levels of ROS are a well-known cause of oxidative damage in eye disorders including age-related macular degeneration (AMD) and uveitis [20]. To the contrary, this hazardous effect of ROS is an important component of the innate immune defense response of immune cells and is utilized to cope with infectious pathogens from the outside environment. In phagocytes, the rapid ROS production, which is termed “oxidative burst”, is one of the earliest cellular responses to infection. Apoplastic generation of superoxide (O_2_^−^) or its dismutation product hydrogen peroxide (H_2_O_2_) can kill invading *T. gondii in vitro* and *in vivo* [21]. A variety of nonphagocytic cells also possess superoxide-producing enzymes, which generate low levels of ROS in response to various extracellular stimuli [22], [23]. Notable, RPE itself and its neighboring photoreceptors produce considerable ROS, because of photochemical reactions that converts light energy to chemoelectrical signals [24].

The sources of ROS include NADPH oxidases, the mitochondria electron transport chain, xanthine oxidase, and endothelial nitric oxide synthase [23], [25]. Although these enzymes contribute to the oxidative burden, evidence suggests that NADPH oxidase is the main enzymatic source of ROS production in human RPE cell lines [26]. Recent studies have demonstrated that NADPH-dependent oxidative stress induces apoptotic signaling pathways in RPE cells and that NADPH oxidase is mechanistically involved in the pathogenesis of some types of retinal degeneration *in vivo* [27]. NADPH oxidase activity occurs in all cells of the vascular wall, but the distribution of the subunits of this enzyme is cell-specific. The family of NADPH oxidases (Nox/Duox) consists of Nox1, Nox2 (gp91phox), Nox3, Nox4, and Nox5 as well as Duox1 and Duox2 [23].

In the present study, we provide evidence that activation of the host cell PI3K/Akt pathway by *T. gondii* infection contributes to the replication of this parasite. Nox4 is the primary source of sustained ROS in RPE cells and thereby is the main target used by *T. gondii* to disarm the oxidative immune system of the host cell for its benefit.

## Materials and Methods

### Reagents and Antibodies

Akt and Phosphospecific Akt (Ser473) antibodies were obtained from Cell Signaling Tech., Inc. (Beverly, MA, USA). Anti-Nox4 was obtained from Santa Cruz Biotechnology (Santa Cruz, CA, USA). Monoclonal anti-actin, HRP-conjugated anti-rabbit and anti-mouse secondary antibodies were purchased from Jackson ImmunoResearch Laboratories (West Grove, PA, USA). PI3K inhibitors, LY294002 and wortmannin, were obtained from Sigma (St. Louis, MO, USA) and PI3K-specific inhibitors, GDC-0941 and ZSTK474, were purchased from Selleck Chemical. Co.

### Cell Culture

Human retinal pigment epithelial cells, ARPE-19 (ATCC, Manassas, VA, USA), were cultured in a 1∶1 mixture of DMEM and nutrient mixture F12 (DMEM/F12) supplemented with 10% heat-inactivated FBS and antibiotic–antimycotic reagents (all from Life Technologies Corporation, CA, USA). One day before treatment, the culture medium was changed to DMEM/F12 medium without FBS.

### Parasites

Tachyzoites of *T. gondii* RH strain that expresses transgenic green fluorescent protein (GFP-RH) were kindly provided by Dr. Yoshifumi Nishikawa (Obihiro University of Agriculture and Veterinary Medicine, Japan). ARPE-19 cells were infected with the GFP-RH strain of *T. gondii* (parasite:cell ratio = 5∶1) and incubated at 37°C and 5% CO_2_ for 2–3 days. Following spontaneous host cell rupture, parasites and host cellular debris were washed in cold PBS. After centrifugation, the final pellet was resuspended in cold DMEM/F12, and the resuspension was passed through a 26-gauge needle and a 5.0-µm pore filter (Millipore, Billerica, MA, USA).

### Preparation of ESP

Purified tachyzoites (3 × 10^8^) were incubated at 37°C for 1 hr under mild agitation in 0.5 ml Hank’s balanced salt solution (HBSS) (Gibco BRL, Rockville, MD, USA). After centrifugation for 5 min at 6,000 rpm, the supernatant containing ESP was saved. The protein concentration was determined with Bradford assay method using BSA as the standard, and samples were stored at −70°C until use.

### Mycoplasma and Endotoxin Detection Assay

To assess mycoplasma and endotoxin levels in the different samples, commercial EZ-PCR Mycoplasma Test kit (Biological Industries, Beit Haemek, Israel) and quantitative chromogenic method (Pierce LAL (*Limulus* amebocyte lysate) Chromogenic Endotoxin Quantification Kit (Thermo Scientific Co, PO)) were used. The *Limulus* amebocyte lysate contains an enzymatic system that is activated in the presence of endotoxin. The lower limit of detection of this assay is 0.1 EU/ml. No contamination of mycoplasma or endotoxin was detected in cell or parasite culture systems (Fig. S1).

### Immunoblot

ARPE-19 cells were grown in 6-well plates and were serum starved overnight to remove any stimulation from serum factors. Cells were infected with tachyzoites or treated with ESP as indicated. After the cells were washed with ice cold PBS, proteins were extracted with PRO-PREP Protein Extraction Solution (iNtRON Biotechnology, Korea) and then incubated on ice for 20 min followed by centrifugation for 5 min at 13,000 rpm at 4°C. Equal amount of protein from each sample were loaded onto a 12% SDS-PAGE gel and separated by electrophoresis. Proteins were then transferred to a polyvinylidene fluoride (PVDF) membrane. Membranes were blocked with 5% nonfat skim milk in TBS containing 0.1% Tween 20 (TBST) for 1 h at room temperature. After being washed once in TBST, membranes were incubated with primary antibody diluted in TBST supplemented with 5% BSA for 2 h at room temperature or overnight at 4°C. After three washes with TBST, membranes were incubated with HRP-conjugated secondary antibody (Jackson ImmunoResearch Laboratories, Inc., USA) for 2 h at room temperature. After three washes with TBST, protein bands were visualized by an ECL chemiluminescence kit (Amersham Biosciences, Freiburg, Germany) according to the manufacturer’s instructions. The bands were scanned and quantified using an imaging densitometer (Bio-Rad Laboratories, Inc., Hercules, CA, USA).

### Proliferation of *T. gondii*

ARPE-19 cells were cultured in 24-well plates containing glass coverslips and serum-starved overnight prior to infection with *T. gondii* at a multiplicity of infection (moi) of 5. Some sets of cells were pretreated with PI3Kinase inhibitors for 1 h and then rinsed 3 times with PBS before infection with *T. gondii*. In order to “**synchronize**” the *T. gondii* replication starting point, we have designed very narrow window for *T. gondii* infection (2 h infection then wash off free-, uninfected-*T. gondii*). By this process, we were also able to eliminate the side effect from secreted ESP of free-, uninfected *T. gondii* and possible invasion rate difference can be ignored, because the proliferation rate of only “infected” *T. gondii* was measured.

After 24 h of incubation, the coverslips were washed with PBS and then fixed with 4% formaldehyde. Cells were stained with Texas Red®-X phalloidin (Life Technologies Corporation, CA, USA) to label F-actin and mounted on slides using mounting medium with DAPI (Vector Laboratories, USA). Cells were then imaged using fluorescence microscopy or confocal microscopy. One to two hundred PVs were randomly selected in each preparation, and parasite replication was monitored by counting the number of tachyzoites per PV. Three separate experiments were performed for statistical analysis of the results.

### Fluorescence Microscopy

ARPE-19 cells were cultured in 24-well plates containing glass coverslips overnight and then infected with GFP-RH tachyzoites for 23 h or with ESP for 1 h. Cells were then exposed to 100 µM H_2_O_2_ (Sigma Chemical Co) for 1 h. Following washing with HBSS, cells were incubated with 10 µM ROS-sensitive dye dihydroethidium (DHE; Life Technologies Corporation) for 30 min at 37°C in the dark. The stained cells were imaged using an Olympus BX-51 fluorescence microscope. All images of the DHE staining were taken using the same exposure time.

### Flow Cytometry

Cell were cultured in 6-well dishes and treated with ESP according to the experimental design. The cultured cells were stained with 10 µM DHE and washed with HBSS. Cells were trypsinized and centrifuged. Then cells were resuspended in HBSS and analyzed on a FACS (BD Biosciences, San Diego, CA).

### NADPH Oxidase Expression Analysis by RT-PCR

Total RNA from stimulated cells was prepared using the Trizol reagent (Invitrogen, Carlsbad, CA, USA), and RNA was reverse transcribed using ReverTra Ace RT kit (Toyobo, Osaka, Japan). All PCR reactions were performed with a MyCycler (Bio-Rad) for 25 cycles. The Nox1-4 and p22phox primer sequences for RT-PCR were described by Claudia Piccoli et al. [28]. Nox5 RT-PCR primer sequences were described by Chou et al. [29]. The other primer sequences (5′-3′) are as follows: Duox1 forward, ATCAATCGGAACTCAAGTGTCTC; Duox1 reverse, AACCAACACATGGTCCTCTCG; Duox2 forward, GCTACCATGTTCTTTCCGACG; and Duox2 reverse, GAGTGCGAGGAGCCATAGAT. Amplified products were electrophoresed in a 2% agarose gel and visualized with ethidium bromide. The relative intensity of bands for each mRNA was related to the intensity of the autoradiogram band of the internal control, GAPDH. Quantification of mRNA was performed using an imaging densitometer (Bio-Rad Laboratories, Inc.).

### siRNA Transfection

Two different duplex sequences against human Nox4 were used as Weyemi et al. reported [30]. Cells were transfected with siRNA duplexes specific for human Nox4, using LipofectamineTM RNAiMAX (Life Technologies Corporation) according to the manufacturer’s protocol. Briefly, cells were seeded in 6-well plates, grown for 24 h (70% confluence), and then transfected with 6–24 nM Nox4-specific siRNA or negative control siRNA (Santa Cruz, CA) for 48 h. Then, cells were infected with *T. gondii* for 24 hours, and then *T. gondii* proliferation was evaluated. The knockdown efficiency was determined by RT-PCR and western blot.

### Statistical Analysis

The data were analyzed using GraphPad Prism data analysis program. For the comparison of statistical significance between two groups, independent sample Student’s *t*-test was used. For multiple comparison, one-way ANOVA followed by *post hoc* comparisons of the group means according to the method of Tukey was used. *P*-values <0.05 were considered significant.

## Results

### Activation of Host PI3/Akt by *T. gondii* Infection and ESP Treatment

As an intracellular parasite, *T. gondii* relies on the growth environment provided by the host cell. The serine/threonine kinase Akt plays a central role in growth regulation of cells, and its activity is regulated by phospholipid binding and activation phosphorylation at Thr308 by PDK1 and by phosphorylation within the carboxy terminus at Ser473 [31]. To identify intracellular signaling mechanisms of the host cell that are responsible for the proliferation of *T. gondii*, we determined whether *T. gondii* infection induces Akt phosphorylation in ARPE-19 cells using immunoblot analysis with phosphospecific Ser473 Akt antibody. In addition, the effects of *T. gondii* ESP on Akt signaling were examined as well. ESP plays an essential function to provide appropriate environment for the entry of the parasite into host cell [11].

**Figure 1:**
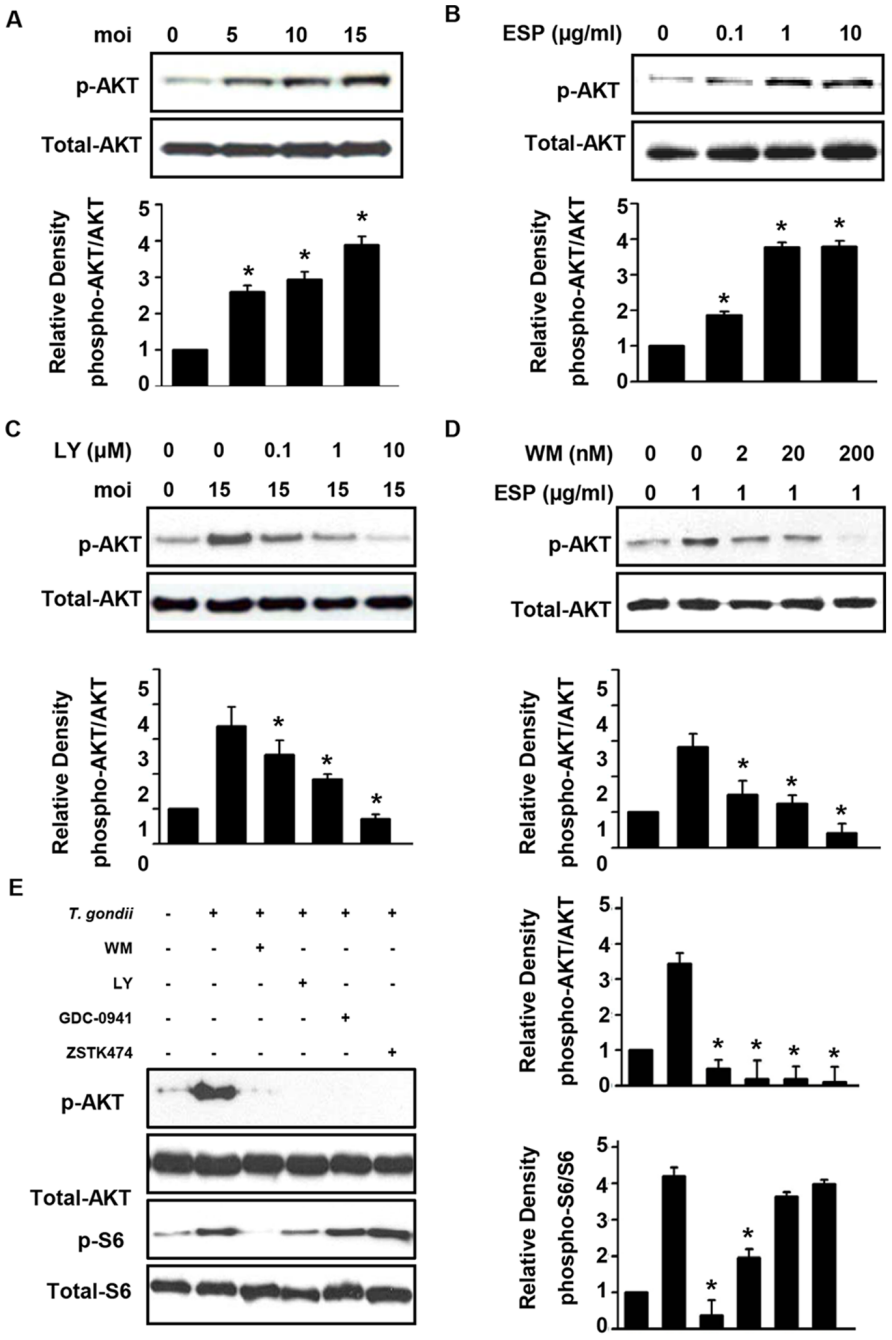
*T. gondii* and ESP activate the PI3K/Akt pathway in ARPE-19 cells. ARPE-19 cells were deprived of serum for 1 day before use in subsequent experiments. (A) Cells were infected with GFP-RH tachyzoites at the indicated moi for 24 h. Two hours after infection, the extracellular tachyzoites were immediately removed by washing with PBS and then incubated in the absence of serum media for 22 h. (B) Cells were stimulated for 1 h with various concentrations of GFP-RH tachyzoites ESP ranging from 0.1 to 10 µg/ml. (C) ARPE-19 cells were infected with *T. gondii* (moi 15) for 24 h and treated with increasing concentrations of LY294002 for the final 1 h. (D) Cells were pretreated with wortmannin for 1 h, and then after washing, cells were stimulated with 1 µg/ml ESP for 1 h. Whole cell lysates were collected and analyzed by western blot using primary antibodies against phospho-Akt and total Akt. Akt phosphorylation levels were calculated as the ratio of untreated control cells after normalization to the amount of total Akt. (E) Cells were infected at moi of 15 for 23 h and then treated with either inhibitor for 1 h. Values represent the mean ± SD values of triplicates. Data are representative of three independent experiments. * denotes *p*<0.05 and indicates that differences were considered significant.

To minimize basal Akt activity, these experiments were performed in serum-starved cells. After *T. gondii* infection for 24 h or ESP stimulation for 1 h, cells were collected for immunoblot analysis to measure host Akt activation. The ratios between the intensities of phosphorylated Akt and total Akt in the infected and stimulated samples compared with those in the uninfected or unstimulated samples as determined by densitometric analysis are shown in Fig. 1A and, B. Phosphorylation of Akt occurred in cells infected with *T. gondii* for 24 h, and host Akt activity was significantly increased according to the number of *T. gondii* per infected cell. ESP also increased Akt phosphorylation in a dose-dependent manner, and this finding is consistent with the *T. gondii* infection experiment. Host cell ciability was tested with MTT assay and proved that viability of ARPE-19 was not significantly reduced by *T. gondii* infection (moi 15) after 24 h (Fig. S2A).

As *T. gondii* infection resulted in Akt phosphorylation in the host ARPE-19 cell, we analyzed the impact of *T. gondii* infection or ESP treatment on an upstream component of Akt signaling. Wortmannin and LY294002 are well-known inhibitors for PI3K, which is the upstream activator of Akt. We examined whether blocking host cell PI3K activation results in inhibition of *T. gondii*-induced Akt phosphorylation. ARPE-19 cells were infected with *T. gondii* for 24 h and treated with LY294002 during the final 1 h. Indeed, sequential increases in LY294002 concentration led to inhibition of the *T. gondii* infection-induced Akt phosphorylation (Fig. 1C). In a similar manner, pretreatment with wortmannin blocked Akt phosphorylation induced by *T. gondii* ESP treatment (Fig. 1D). Although wortmannin and LY294002 are well-known inhibitor for PI3K, it is also known that they are not specific to PI3K and can inhibit mTOR pathway. In order to confirm that PI3K/Akt pathway is indeed involved in *T. gondii* induced events, GDC-0941 and ZSTK474 were tested for their effect on *T. gondii* induced host cell response, GDC-0941 and ZSTK474 successfully inhibited Akt phosphorylation by *T. gondii* in a dose-dependent manner (Fig. S3) but S6 phosphorylation which is under control of mTOR was not affected (Fig. 1E). These results suggest that *T. gondii* and ESP activate host Akt in a PI3K-dependent manner.

### Roles of Akt/PKB Signaling in Intracellular *T. gondii* Proliferation in the Host

**Figure 2:**
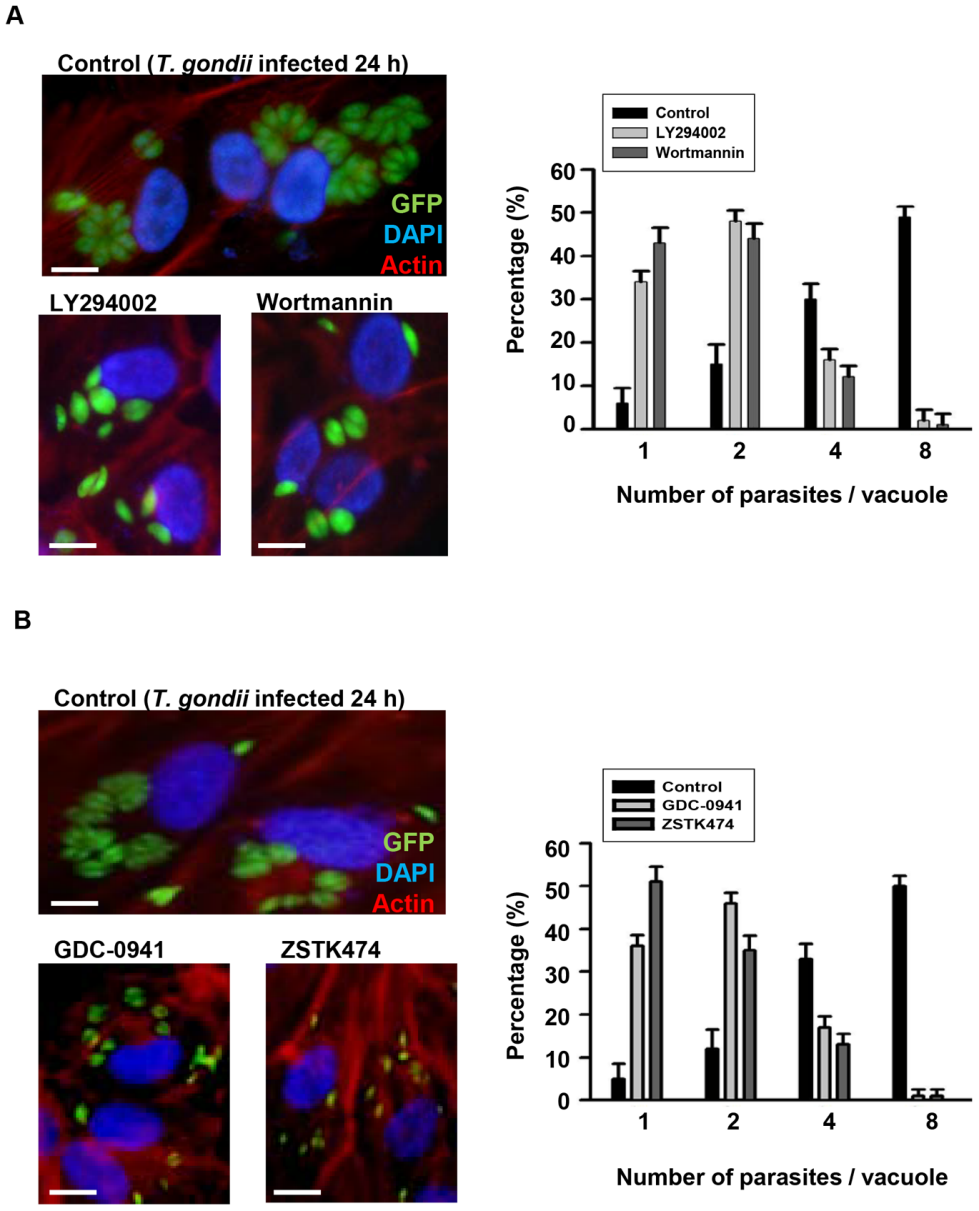
PI3K inhibitors inhibited *T. gondii* proliferation in ARPE-19 cells. The proliferation of tachyzoites in ARPE-19 cells was examined by fluorescence microscopy. Cells were pre-incubated with PI3K inhibitors, either 10 µM LY294002, 200 nM wortmannin (A), or 250 nM GDC-0941, 10 nM ZSTK474 (B) for 1 h. After washing, the cells were then infected with *T. gondii* at moi of 5 for 24 h. Cells were fixed and stained with Texas Red®-X phalloidin for labeling F-actin (red), and nuclei were stained with DAPI (blue). The number of parasites per vacuole were counted and converted to percentage. Data are representative of three independent experiments. Scale bar = 100 µM.

Several independent lines of evidence indicate that *T. gondii* infection renders cells resistant to proapoptotic stimuli [32]. This effect is presumed to be beneficiary to *T. gondii* survival and proliferation in the host cell. Following phosphorylation by activating kinases, Akt affects multiple cellular targets that increase metabolism, growth, and synthetic processes and suppress apoptosis [33]. *T. gondii*, as an intracellular parasite, relies on the growth environment provided by the host. As a result, we initially investigated the effect of PI3K inhibitors on *T. gondii* proliferation in ARPE-19 cells. Statistically significant inhibition of *T. gondii* growth and proliferation by LY294002 and wortmannin was detected (Fig. 2). The numbers of tachyzoites in ARPE-19 cells, which had been pre-incubated with PI3K inhibitors for 1 h, washed, and then infected with *T. gondii* at an moi of 5, were counted 24 h post-infection. Without inhibitor pretreatment, most tachyzoites divided two times to generate four parasites per PV. PI3K-specific inhibitors, GDC-0941 and ZSTK474, also showed similar effect as LY294002 and wortmannin on *T. gondii* proliferation that inhibition of the PI3K/Akt pathway reduced the rate of *T. gondii* division, and most PVs only contained one or two *T. gondii*. Proliferation rate observation at different time point (12 h) also showed that PI3K inhibitors delay the proliferation of *T. gondii* compared to untreated (Fig. S2B). These results suggest that *T. gondii*-induced PI3K/Akt signaling is important for *T. gondii* proliferation in the host cell.

Finally, given that the presence of mycoplasma and endotoxin could affect PI3K signaling and response to ROS, we quantified the levels of mycoplasma and endotoxin in the culture medium, cell, parasite and ESP. The results were shown in Figure S1 that no contamination detected in our experimental systems.

### T. gondii-induced Activation of Host PI3K/Akt Signaling Pathway Reduced Intracellular ROS Levels of ARPE-19 Cells Under Oxidative Stress

**Figure 3:**
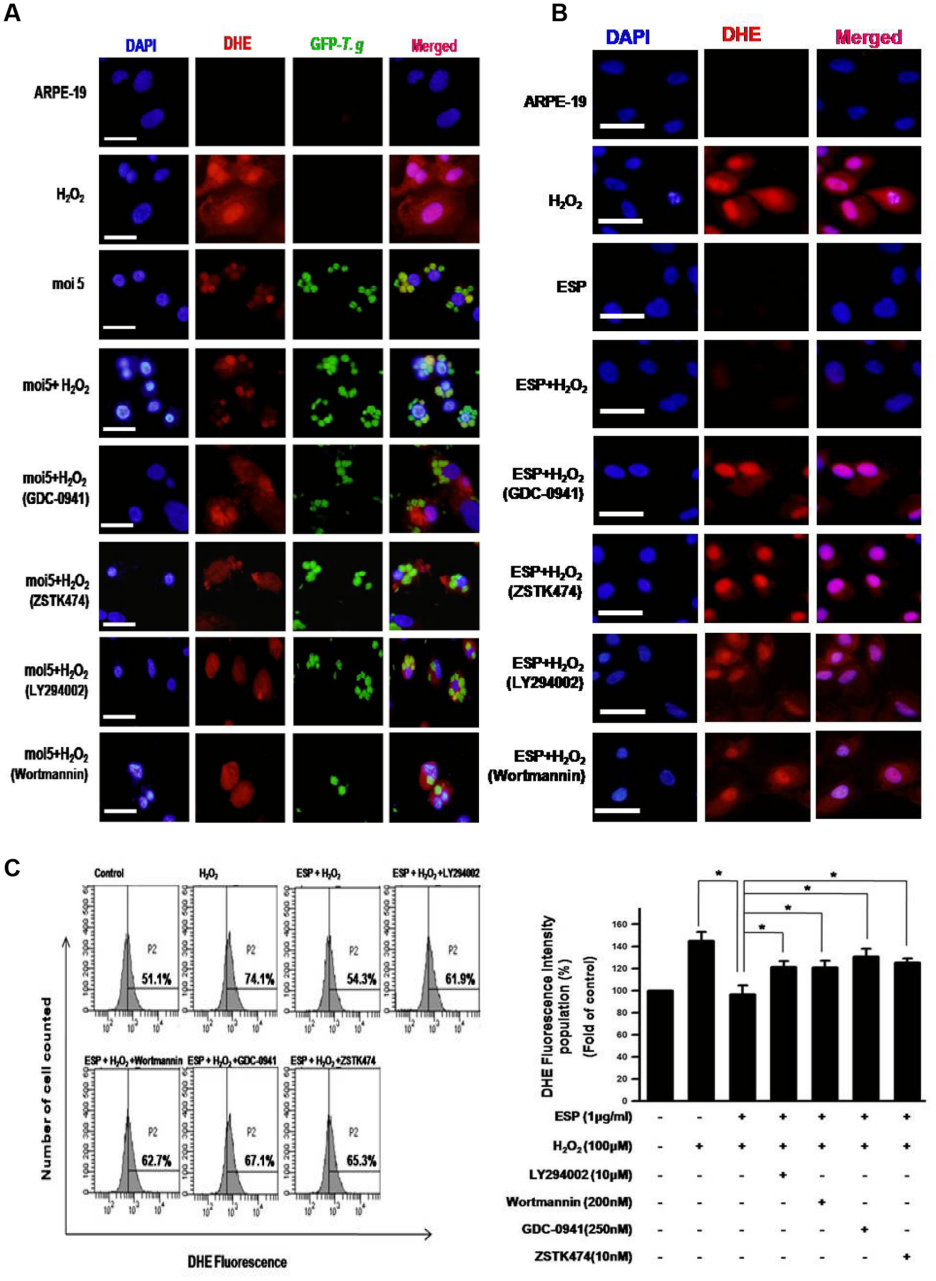
*T. gondii* and ESP inhibited H_2_O_2_-induced ROS generation via activation of PI3K/Akt signaling. Fluorescence microscopy images of cells stained with 10 µM DHE and treated with different conditions. ARPE-19 cells were infected with GFP-RH for 23 h (A, B), or ARPE-19 cells were stimulated with ESP for 1 h (C, D). These cell were exposed to 100 µM H_2_O_2_ with or without inhibitor for 1 h. The ROS level change by ESP and PI3K inhibitors under oxidative stress (H_2_O_2_) were quantitatively analyzed by flow cytometry (E). Results are representative of three independent experiments. * denotes *p*<0.05, and these differences were considered significant. Scale bar = 100 µM.

The host cell generates various threats to attack parasite infection, and production of ROS is one very effective way to kill the infiltrators [21]. Since the PI3K/Akt pathway is involved in cell survival signaling and is important for *T. gondii* proliferation, we studied the effect of host PI3K/Akt signaling induced by *T. gondii* against H_2_O_2_ oxidative stress in ARPE-19 cells. ARPE-19 cells were stimulated with 100 µM H_2_O_2_ and then labeled with the cell-permeable fluorescent dye DHE as a cytosolic superoxide indicator. Intracellular levels of ROS were analyzed by fluorescent microscopy. The positive control, which received only H_2_O_2_ treatment, exhibited high DHE fluorescent signal. However, *T. gondii* infection or ESP treatment markedly reduced the host cytosolic ROS even in the presence of H_2_O_2_ treatment (Fig. 3A, B). Intriguingly, additional treatment with the PI3K inhibitors wortmannin, LY294002, GDC-0941 or ZSTK474 blocked the reduced ROS level induced by *T. gondii* infection or ESP treatment (Fig. 3A, B).

Additionally, the flow cytometry were performed to quantitatively analyze the effect of ESP and PI3K inhibitors on host intracellular ROS level after H_2_O_2_ treatment (Fig. 3C). Flow cytometrical analysis with *T. gondii* infected cell was excluded because it is found that *T. gondii* itself can be strongly labeled by DHE (Fig. 3 and Fig. S4), and it interfered the reading the fluorescence intensities of DHE labeled- host cell cytoplasm by FACS. These results suggest that activation of host Akt/PKB signaling by *T. gondii* provides a protective function for infected *T. gondii* by blocking the ROS-dependent host defense system.

### *T. gondii* ESP-induced Activation of Host PI3K/Akt Signaling Pathway Reduced Basal Intracellular ROS Levels of ARPE-19 Cells

**Figure 4:**
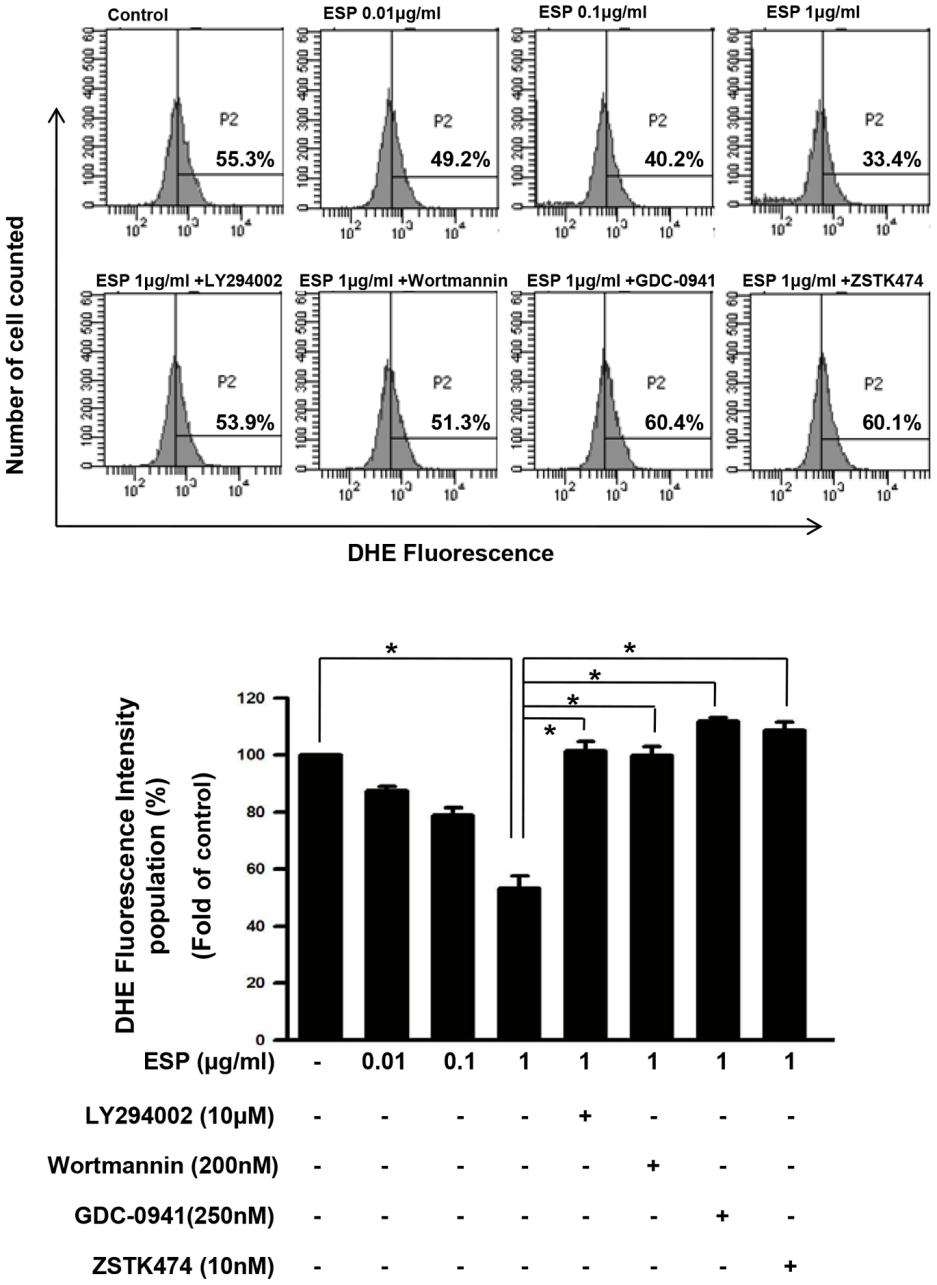
*T. gondii* ESP decreased endogenous ROS levels in ARPE-19 cells. (A) Cells were stimulated with ESP for 1 h and then incubated with PI3K inhibitors for 1 h. Intracellular superoxide was measured by DHE staining followed by flow cytometry. (B) Relative fluorescence intensity of the population of DHE-stained cells corresponding to *A*. The results are expressed as mean ± SD of three independent experiments. * denotes *p*<0.05, and these differences were considered significant.

Next, we tested whether ESP can reduce the basal ROS level of host cell (H_2_O_2_ untreated) into even lower level. Because it is reported that oxidative stress in ARPE-19 cells can activates mTOR signaling pathway [34], we wanted to test the ROS-reducing ctivity of ESP under the condition which possible side effect of H_2_O_2_ is minimized. Flow cytometric analysis indicated an approximately 50% decrease in ROS levels 5 h after 1 µg/ml ESP treatment (Fig. 4). This result was in agreement with the fluorescent microscopy analysis, indicating that the host intracellular ROS decreased markedly after *T. gondii* infection (Fig. 3B).

Next, we examined whether PI3K inhibitors blocked the ROS reduction by *T. gondii* ESP treatment in ARPE-19 cells. As expected from the previous results, the decrease in ROS induced by *T. gondii* ESP 5 h after treatment was almost completely reversed by incubation with LY294002, wortmannin, GDS-0941 or ZSTK474 (Fig. 4). Taken together, these data indicate that infection with *T. gondii* stimulates host PI3K/Akt signaling to suppress ROS generation and that this process may play an important role in the survival and proliferation of infected *T. gondii*.

### *T. gondii* Targets Nox4 to Decrease Host ROS Levels via Activation of PI3K/Akt Signaling

**Figure 5:**
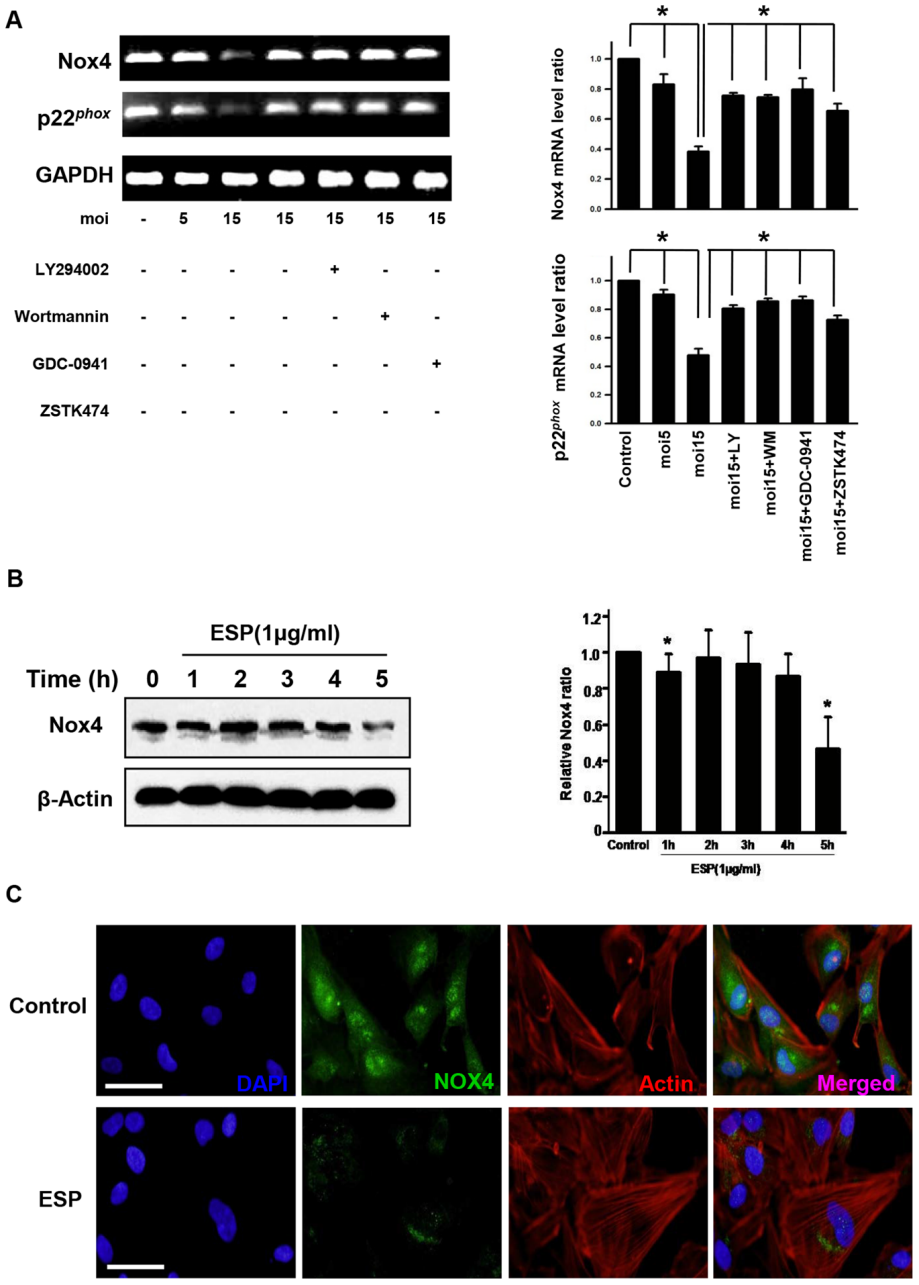
*T. gondii* and ESP suppressed host Nox4 expression via the PI3K/Akt pathway. (A) RT-PCR analysis of the host Nox4 and P22^phox^ mRNA levels in ARPE-19 cells. GAPDH was used as an internal control. (B) Cells were stimulated with 1 µg/ml ESP for the indicated times. Nox4 protein levels were analyzed by western blot, and β-actin was used as a loading control. (C) Immunocytochemistry of Nox4 in the ESP-stimulated cells. ARPE-19 cells were stimulated with 1 µg/ml ESP for 5 h. Cells were subsequently stained with anti-Nox4 antibody (green) and Texas Red®-X phalloidin for labeling F-actin (red). Nuclei were stained with DAPI (blue). The results are expressed as mean ± SD of three independent experiments. Scale bar = 100 µM. * denotes *p*<0.05, and these differences were considered significant.

Since *T. gondii* infection activated PI3K/Akt signaling and intracellular ROS levels were significantly reduced under both unstressed conditions and oxidative challenge, we sought to identify the ROS generator that is under control of PI3K/Akt signaling. Our experiments indicated the presence of a major oxidase to generate ROS, which may suppress *T. gondii* replication in host cells. To explore this idea, the mRNA levels of Nox/Duox family of ROS-generating NADPH oxidases were examined in ARPE-19 cells by RT-PCR. Nox4 was abundantly expressed in ARPE-19 cells, whereas the expression levels of the other Nox and Duox oxidases were undetectable (Figure S5). Interestingly, expression of Nox4 and its functional partner p22^phox^ were suppressed by *T. gondii* infection in a moi-dependent manner. Also, PI3K inhibitor treatment experiment revealed that Nox4 gene expression was regulated by the PI3K/Akt signaling pathway (Fig. 5A). To confirm that the Nox4 protein level was decreased in agreement with the decrease in its mRNA level, immunofluorescence and immunoblot analysis were performed using anti-Nox4-specific antibody. Indeed, the Nox4 protein level was significantly reduced after *T. gondii* ESP treatment for 5 h (Fig. 5B, C). These findings demonstrated that *T. gondii* specifically suppresses host Nox4 gene expression via the PI3K/Akt signaling pathway in ARPE-19 cells.

### Nox4 Expression is Important for *T. gondii* Intracellular Replication

**Figure 6:**
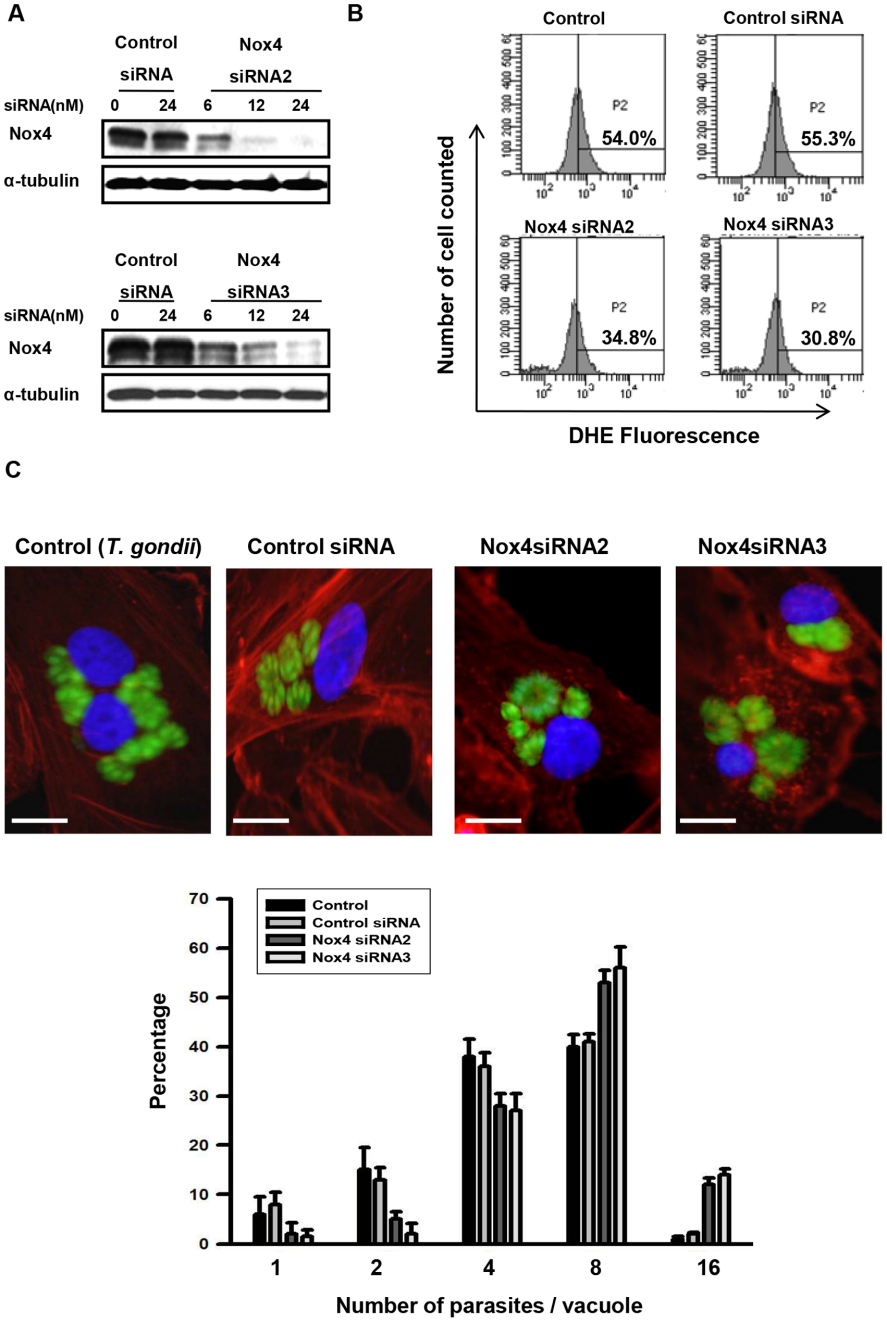
Nox4 gene knockdown promoted *T. gondii* replication. ARPE-19 cells were transfected with *Nox4*-specific siRNA for 48 h. (A) Nox4 knockdown efficiency was determined by western blot. (B) Intracellular ROS levels were measured by flow cytometry after DHE staining. (C) ARPE-19 cells transfected with control siRNA or specific *Nox4* siRNAs were infected with *T. gondii* for 24 h, and fluorescence micrographs of intracellular tachyzoite replication 24 h post-infection were obtained. The results are expressed as mean ± SD of three independent experiments.

Experiments using specific siRNA duplexes to silence Nox4 expression in ARPE-19 cells were performed to determine whether Nox4-dependent production of ROS is important for intracellular replication of *T. gondii*. A series of candidate siRNA molecules was transfected into ARPE-19 cells for 48 h, and Nox4 protein levels were determined by immunoblot using a rabbit antibody raised against Nox4 (Fig. 6A). Both siRNA2 and siRNA3 (24 nM) consistently resulted in a 75–85% decrease in Nox4 protein expression, and thus, these siRNAs were selected for use in subsequent experiments. Intracellular ROS levels in ARPE-19 cells were significantly reduced by more than 40% following transfection with Nox4 siRNA, demonstrating that Nox4 plays a important role in the production of ROS in ARPE-19 cells (Fig. 6B).

To determine the role of Nox4 in *T. gondii* proliferation, ARPE-19 cells, which were incubated 48 h after transfection with Nox4 siRNA, were infected for another 24 h with *T. gondii*, and the proliferation of *T. gondii* was evaluated under fluorescence microscope (Fig. 6C). In untranfected cells and in control siRNA treated cells, the PVs with 8 tachyzoites are approximately 40% of total PV population; however, in Nox4 siRNA-transfected cells, more than 50% of PVs contained 8 tachyzoites and even PVs with 16 tachyzoites (approx 10%) were observed after 24 h infection, demonstrating accelerated replication of *T. gondii* in these cells. These results indicated that infection with *T. gondii* suppresses the gene expression of Nox4 in host cells to reduce ROS generation and to generate a favorable intracellular environment for *T. gondii* growth and proliferation.

## Discussion

It is common to find cysts and tachyzoites in the retina of *T. gondii* infected patients. Necrotic lesions are often observed which come with degeneration of the neuronal retina and choroids structure (retinochordoiditis) [35]. Mouse model studies have reported that retinal lesions are the results of inflammation reaction caused by mononuclear cells at the site of infection. Although, *T. gondii* favorably infiltrate and reside in the eye structures, for example, retinal pigment epithelium (RPE) to show dramatic symptoms, it is known that eye is an immune privileged organ which is protected from immune mediated inflammation and damage by various mechanisms [36]. The cells of the retinal pigment epithelium play important role in the physiology of the retina by recycling light-induced all-*trans*-retina from photoreceptor cells into 11-*cis*-retina and providing functional photopigments back to photoreceptors, and by eliminates oxidatively damaged materials from photoreceptors [24]. This means that RPE cells are basically prone to oxidative stress and capable to produce reactive oxygen species under certain condition [37]. For example, exposure to bright light may generate free radical and increase ROS production, which further induces oxidative stress in RPE cells [38]. Based on these facts, we tried to reveal how *T. gondii*’s survived and proliferate in such harsh condition.

*T. gondii*, like many other pathogens, forms a membranous vacuole to reside in when it infiltrates into host cell in order to avoid lysosomal attack from the host cell. The parasitophorous vacuolar membrane (PVM) itself works as physical barrier but also in order to increase their survivability, various signal related events occurs around the PVM. It has been reported that ADP-ribosylation factor-6 (ARF6) activate PI3K and recruit PIP(2) and PIP(3) to parasitophorous vacuole of invading parasites [39]. Other studies also showed that upon infection with *T. gondii*, host PI3K/Akt pathway was activated, and this activation was required for host cell resistance to apoptosis [32], and Akt activation was also required for *T. gondii* to coordinate control of host centrosome position, organelle distribution, and migratory response [40]. These reports suggest that host PI3k/Akt pathway is one major target for *T. gondii* to modify and utilize for its own beneficial events.

Our new finding is that PI3K/Akt signaling pathway activation is essential for intracellular *T. gondii* proliferation. In order to examine the intracellular tachyzoite replication rate, the replication number of GFP-labeled-*T. gondii* in each PV was monitored by fluorescent microscopy. This method was also useful to exclude the multi-infected *T. gondii* and count exact number of each *T. gondii* in each PV (More useful than FACS which just measuring the intensity of GFP to evaluate the number of *T. gondii*).

Most of intracellular tachyzoites divides twice to generate four individuals 24 h after infection. Inhibition of PI3K/Akt pathway by pre-treatment of LY294002 and wortmannin significantly reduced its dividing rate that *T. gondii* replication occurred only once after 24 h infection to generate two parasites in one PV compare to untreated control groups. These data suggest that PI3K/Akt signaling pathway activation by *T. gondii* is important for its proliferation against host innate immune defense system.

GDC-0941 inhibits all class 1 PI3K isoforms but does not significantly suppress the activity of any other lipid or protein kinase tested including mTOR [41]. ZSTK474 is known as an ATP-competing inhibitor of PI3K, and the inhibitory activity of ZSTK474 is even isoform-specific [42]. To the contrary, it is reported that even at a concentration of 100 µM, ZSTK474 inhibited mTOR activity rather weekly. The unique properties of GDC-0941 and ZSTK474, specific inhibition on PI3K activity but non-inhibitory effect on mTOR, were also confirmed by ourselves (Fig. 1E) and the experiments with these PI3K- specific inhibitors provided us more confidence on our hypothesis that PI3K/Akt pathway rather than mTOR pathway is important in regulation of host ROS and later Nox4 expression by *T. gondii*.

For the next step, we focused on unknown, underlying host mechanism which might hinder the infected *T. gondii* from its growth. It has long been known that ROS plays essential role in innate immune responses against pathogens, such as bacteria or intracellular parasites, through the production of superoxide by reduced nicotinamide adenine dinucleotide phosphate (NADPH) oxidase. Because of that, in the view of intracellular parasites, reducing, removal or intoxicating the intracellular ROS is the urgent requirement for their survival and proliferation [20]. It has been reported that proliferation of *T. gondii* can diminish oxygen radical production in the macrophage cell *in vivo*, but the most of the mechanisms and pathways related to the event is not fully understood yet [43].

Our data clearly showed that not just *T. gondii* only, but ESP also can reduce the host ROS level in the presence or absence of oxidative stress stimuli condition. Surprisingly, treatment of PI3K inhibitors significantly suppressed the *T. gondii* or ESP activity to reduce host ROS level. By combining these two results, we were able to deduce a hypothesis that for the *T. gondii* proliferation in RPE, ROS level reduction in host cell is important and this environmental regulation is achieved by activating host PI3K/Akt signaling pathway.

When we started the *T. gondii*-involved host ROS regulation study, it was our plan to combine data from immunocytochemistry and FACS analysis was used in order to cross-check the results from each experiments, respectively. Though, *T. gondii* itself contains large amount of ROS inside and *T. gondii* infected cell was therefore, not suitable for FACS analysis. In order to detour the problem, treatment of ESP to ARPE cell, instead of infecting live *T. gondii*, was established as a very useful model system to study the action of parasite infection on host ROS level modulation.

Next, all these data strongly suggests that *T. gondii* induced signal activation has specific target to reduce the generation of ROS in host ARPE19 cell. As described in introduction, mammalian cells have developed many ROS generating enzymes during the evolution process and NADPH oxidases have been known as one of the major player for ROS production in RPE and other mammalian cells. The family of NADPH oxidases (Nox/Duox) consists of Nox1-5, as well as the Duox1 and Duox2 [33]. Among these candidates, in order to identify a NADPH oxidase which is regulated by PI3k/AKT signaling pathway, first, we checked the mRNA expression level of each enzymes in ARPE19 cells. Surprisingly, Nox4 turned out to be the only NADPH oxidase which has significant amount of mRNA transcript in ARPE19 cell (Fig. S5). Other NADPH oxidases, including the Nox2, a well-known inducible NADPH oxidase in macrophage, did not show any change in its level even after *T. gondii* infection. Instead, *T. gondii* infection caused significant reduction of Nox4 mRNA and protein level, and intracellular ROS level of the host. These results suggest that Nox4 is the main target for *T. gondii* in reducing host intracellular ROS level, and PI3K/Akt signaling pathway is responsible for the suppression of Nox4 expression. Another interesting finding is that P22*^Phox^* transcription was suppressed by *T. gondii* infection. P22*^Phox^* is a required component for NADPH oxidases to express full enzymatic activity and stabilization of the enzyme complex. For example, in phagocytes, a well-characterized mulitcomponent enzyme, in which the catalytic subunit gp61/Nox2 and P22*^Phox^* form an integral complex called flavocytochrome b558 [23]. Not surprisingly, some pathogens have developed strategies to counter the Nox2 response by either inhibiting Nox2 assembly on the phagosome, as in the case for Salmonella typhimurium [44] and Helicobacter pylori [45], or reducing steady-state level of Nox2 components as illustrated by Anaplasma phagocytophilia [46] or Ehrlichia caffeensis [47].

However, there is a report that forms of P22*^Phox^* mutated in the proline-rich region inhibited ROS production for Nox1, Nox2, and Nox3, but not for Nox4 [23]. There are other reports that Nox4 does not require other components, such as P40*^Phox^*, P47*^Phox^*, P67*^Phox^*, or Rac, for its activation [48]. Given the fact that Nox4 generates substantial amounts of ROS without a need for cell stimulation and cofactors, it implies that *T. gondii* has developed a unique mechanism to suppress the ROS generation by elimination of constantly active Nox4 at the transcription level rather than by regulating its activity in RPE. Down-regulation of P22*^Phox^* transcription level by *T. gondii* would be a sub-system for tissues with traceable amount or inducible Nox enzyme(s) inside. This question would need further study. Silencing Nox4 expression with Nox4 siRNA demonstrated that down-regulation of Nox4 transcription is sufficient enough to reduce the intracellular ROS level and as a consequence, it was observed that in Nox4 siRNA treated host cell, *T. gondii* proliferated more rapidly, compare to *T. gondii* in untreated cell or in control siRNA treated cells.

Previous studies have suggested that multiple transcription factors, such as NF-κB, GATA, AP-1, Ets1, C/EBP, and STAT1/3 are coordinately involved in the regulation of Nox expression and function [49]. These data indicate that cooperation among multiple transcription factors, co-activators, and co-repressors is essential for precise control of Nox transcription and function. Nevertheless, the precise mechanisms of Nox regulation are not entirely defined. Our data demonstrating the involvement of PI3K/Akt pathway in Nox4 transcription imply the possibility that Forkhead Box O (FoxO) transcription factors are need to be added on the list of components which regulate Nox4 transcription. FoxO transcription factors have been associated with redox signaling and regulation of proliferation and apoptosis. These transcription factors share the ability to be inhibited and translocated out of the nucleus on phosphorylation by Akt/PKB in the PI3K signaling pathway. According to our preliminary data, *T. gondii* infection, indeed, induced the phosphorylation of FoxO and relocation of FoxO out of nucleus to cytosol (data not shown) in correlation with intracellular ROS level.

In conclusion, our findings demonstrate that *T. gondii* can manipulate host PI3K/Akt signaling pathway to reduce intracellular ROS level and to generate beneficial microenvironment for their proliferation. According to Nox4 siRNA experiments, it is also indicated that Nox4 is the important component for innate immune defense mechanism of host cell against parasite infection and it is the major target for *T. gondii* to suppress hostile intracellular ROS level. Down-regulation of Nox4 in *T. gondii* infected human RPE cell may represent a novel mechanism of blood-retinal barrier breakdown and pathological retinal in ocular toxoplasmosis.

## Supporting Information

Figure S1 **Examination of Mycoplasma and Endotoxin.** (A) PCR analysis of *ARPE-19 cell and parasite* for detecting the presence of *Mycoplasma* DNA. The size of the PCR product obtained using the positive template with primer pairs is 270 bp. *(B)* Endotoxin levels of *ARPE-19 cell and parasite* detected with the LAL assay.
(TIF)

Figure S2 **Confirmation of host viability after infection of** ***T. gondii*** **and effects of PI3K inhibitors on** ***T. gondii*** **replication ratio in 12 h.** (A) ARPE-19 cells were infected with *T. gondii* at various moi for 24 h, and cell viability was assessed by MTT assay. (B) Cells were pre-incubated with PI3K inhibitors, either 10 µM LY294002, 200 nM wortmannin, or 250 nM GDC-0941, 10 nM ZSTK474 for 1 h. After washing, the cells were then infected with *T. gondii* at moi of 5 for 12 h. The number of parasites per vacuole was counted and converted in to percentage (doubling time of around 10 ∼12 h).
(TIF)

Figure S3 **Effects of PI3K-specific inhibitors on** ***Toxoplasma*** **-induced PI3K/Akt activation.** ARPE-19 cells were infected with *T. gondii* (moi 15) for 24 h and treated with increasing concentrations of GDC-0941 or ZSTK474 for the final 1 h.
(TIF)

Figure S4 **Presence of high ROS in** ***T. gondii.*** *Toxoplasma*-induced ROS synthesis inhibition is suppressed by treatment of PI3K inhibitor. The arrows show that the *T. gondii* can be highly labeled by dihydroethidium (DHE).
(TIF)

Figure S5 **Messenger RNA expression pattern of NADPH oxidases in** ***T. gondii*** **infected ARPE-19 cell.** Reverse transcription-PCR on whole RNA extracted from host cell with primer selected for seven catalytic subunits of NADPH oxidase isoforms (Nox1∼5, Duox1/Duox2).
(TIF)

## References

[1] Hill D, Dubey JP (2002) *Toxoplasma gondii*: transmission, diagnosis and prevention. Clin Microbiol Infect 8: 634–640. 12390281 10.1046/j.1469-0691.2002.00485.x

[2] Kim K, Weiss LM (2004) *Toxoplasma gondii*: the model apicomplexan. Int J Parasitol 34: 423–432. 15003501 10.1016/j.ijpara.2003.12.009 PMC3086386

[3] Coppens I, Joiner KA (2001) Parasitehost cell interactions in toxoplasmosis: new avenues for intervention? Expert Rev Mol Med 2001: 1–20. 10.1017/S1462399401002277 14987366

[4] Black MW, Boothroyd JC (2000) Lytic cycle of *Toxoplasma gondii* . Microbiol Mol Biol Rev 64: 607–623. 10974128 10.1128/mmbr.64.3.607-623.2000 PMC99006

[5] Manea A, Tanase LI, Raicu M, Simionescu M (2010) Transcriptional regulation of NADPH oxidase isoforms, Nox1 and Nox4, by nuclear factor-kappaB in human aortic smooth muscle cells. Biochem Biophys Res Commun. 396: 901–907. 10.1016/j.bbrc.2010.05.019 20457132

[6] Lindmo K, Stenmark H (2006) Regulation of membrane traffic by phosphoinositide 3-kinases. J Cell Sci 119: 605–614. 16467569 10.1242/jcs.02855

[7] Franke TF, Yang SI, Chan TO, Datta K, Kazlauskas A, et al (1995) The protein kinase encoded by the Akt proto-oncogene is a target of the PDGF-activated phosphatidylinositol 3-kinase. Cell 81: 727–736. 7774014 10.1016/0092-8674(95)90534-0

[8] Ehrhardt C, Ludwig S (2009) A new player in a deadly game: influenza viruses and the PI3K/Akt signalling pathway. Cell Microbiol 11: 863–871. 19290913 10.1111/j.1462-5822.2009.01309.x PMC7162392

[9] Wang Y, Karnataki A, Parsons M, Weiss LM, Orlofsky A (2010) 3-Methyladenine blocks *Toxoplasma gondii* division prior to centrosome replication. Mol Biochem Parasitol 173: 142–153. 20609430 10.1016/j.molbiopara.2010.05.020 PMC2917897

[10] MacLaren A, Attias M, de Souza W (2004) Aspects of the early moments of interaction between tachyzoites of Toxoplasma gondii with neutrophils. Vet Parasitol 125: 301–312. 15482886 10.1016/j.vetpar.2004.07.006

[11] Son ES, Nam HW (2001) Detection and characterization of excretory/secretory proteins from *Toxoplasma gondii* by monoclonal antibodies. Korean J Parasitol 39: 49–56. 11301590 10.3347/kjp.2001.39.1.49 PMC2721065

[12] Quan JH, Cha GH, Zhou W, Chu JQ, Nishikawa Y (2013) Involvement of PI 3 kinase/Akt-dependent Bad phosphorylation in *Toxoplasma gondii*-mediated inhibition of host cell apoptosis. Exp Parasitol 133: 462–471. 23333591 10.1016/j.exppara.2013.01.005

[13] Jabs DA (1990) Ocular toxoplasmosis. Int Ophthalmol Clin 30: 264–270. 2228473 10.1097/00004397-199030040-00009

[14] McMenamin PG, Dutton GN, Hay J, Cameron S (1986) The ultrastructural pathology of congenital murine toxoplasmic retinochoroiditis. Part I: The localization and morphology of *Toxoplasma* cysts in the retina. Exp Eye Res 43: 529–543. 3792458 10.1016/s0014-4835(86)80021-5

[15] Tabbara KF, Nozik RA, O’Connor GR (1974) Clindamycin effects on experimental ocular toxoplasmosis in the rabbit. Arch Ophthalmol 92: 244–247. 4136592 10.1001/archopht.1974.01010010252017

[16] Nicholson DH, Wolchok EB (1976) Ocular toxoplasmosis in an adult receiving long-term corticosteroid therapy. Arch Ophthalmol 94: 248–254. 1252177 10.1001/archopht.1976.03910030120009

[17] Mochizuki T, Furuta S, Mitsushita J, Shang WH, Ito M, et al (2006) Inhibition of NADPH oxidase 4 activates apoptosis via the AKT/apoptosis signal-regulating kinase 1 pathway in pancreatic cancer PANC-1 cells. Oncogene 25: 3699–3707. 16532036 10.1038/sj.onc.1209406

[18] Zhang Y, Chen F (2004) Reactive oxygen species (ROS), troublemakers between nuclear factor-kappaB (NF-kappaB) and c-Jun NH(2)-terminal kinase (JNK). Cancer Res 64: 1902–1905. 15026320 10.1158/0008-5472.can-03-3361

[19] Torres M, Forman HJ (2003) Redox signaling and the MAP kinase pathways. Biofactors 17: 287–296. 12897450 10.1002/biof.5520170128

[20] Gritz DC, Montes C, Atalla LR, Wu GS, Sevanian A, et al (1991) Histochemical localization of superoxide production in experimental autoimmune uveitis. Curr Eye Res 10: 927–931. 1659971 10.3109/02713689109020328

[21] Aline F, Bout D, Dimier-Poisson I (2002) Dendritic cells as effector cells: gamma interferon activation of murine dendritic cells triggers oxygen-dependent inhibition of Toxoplasma gondii replication. Infect Immun 70: 2368–2374. 11953372 10.1128/IAI.70.5.2368-2374.2002 PMC127929

[22] Lambeth JD (2004) NOX enzymes and the biology of reactive oxygen. Nat Rev Immunol 4: 181–189. 15039755 10.1038/nri1312

[23] Bedard K, Krause KH (2007) The NOX family of ROS-generating NADPH oxidases: physiology and pathophysiology. Physiol Rev 87: 245–313. 17237347 10.1152/physrev.00044.2005

[24] Kang KH, Lemke G, Kim JW (2009) The PI3K-PTEN tug-of-war, oxidative stress and retinal degeneration. Trends Mol Med 15: 191–198. 19380252 10.1016/j.molmed.2009.03.005 PMC2993245

[25] Landmesser U, Dikalov S, Price SR, McCann L, Fukai T, et al (2003) Harrison Oxidation of tetrahydrobiopterin leads to uncoupling of endothelial cell nitric oxide synthase in hypertension. J Clin Invest 111: 1201–1209. 12697739 10.1172/JCI14172 PMC152929

[26] Qian J, Keyes KT, Long B, Chen G, Ye Y (2011) Impact of HMG-CoA reductase inhibition on oxidant-induced injury in human retinal pigment epithelium cells. J Cell Biochem 112: 2480–2489. 21544860 10.1002/jcb.23173

[27] Chen Y, Okano K, Maeda T, Chauhan V, Golczak M, et al (2012) Mechanism of all-trans-retinal toxicity with implications for stargardt disease and age-related macular degeneration. J Biol Chem 287: 5059–5069. 22184108 10.1074/jbc.M111.315432 PMC3281612

[28] Piccoli C, Ria R, Scrima R, Cela O, D’Aprile A, et al (2005) Characterization of mitochondrial and extra-mitochondrial oxygen consuming reactions in human hematopoietic stem cells. Novel evidence of the occurrence of NAD(P)H oxidase activity. J Biol Chem 280: 26467–26476. 15883163 10.1074/jbc.M500047200

[29] Chou CC, Hsiao HY, Hong QS, Chen CH, Peng YW, et al (2008) Single-walled carbon nanotubes can induce pulmonary injury in mouse model. Nano Lett 8: 437–445. 18225938 10.1021/nl0723634

[30] Weyemi U, Lagente-Chevallier O, Boufraqech M, Prenois F, Courtin F (2012) ROS-generating NADPH oxidase NOX4 is a critical mediator in oncogenic H-Ras-induced DNA damage and subsequent senescence. Oncogene 31: 1117–1129. 21841825 10.1038/onc.2011.327 PMC3307059

[31] James SR, Downes CP, Gigg R, Grove SJ, Holmes AB, et al (1996) Specific binding of the Akt-1 protein kinase to phosphatidylinositol 3, 4, 5-trisphosphate without subsequent activation. Biochem J 315: 709–713. 8645147 10.1042/bj3150709 PMC1217264

[32] Kim L, Denkers EY (2006) *Toxoplasma gondii* triggers Gi-dependent PI 3-kinase signaling required for inhibition of host cell apoptosis. J Cell Sci 119: 2119–2126. 16638808 10.1242/jcs.02934

[33] Ehrhardt C, Ludwig S (2009) A new player in a deadly game: influenza viruses and the PI3K/Akt signalling pathway. Cell Microbiol 11: 863–871. 19290913 10.1111/j.1462-5822.2009.01309.x PMC7162392

[34] Perkins ES (1973) Ocular toxoplasmosis. Br J Ophthalmol 57: 1–17. 4574554 10.1136/bjo.57.1.1 PMC1214816

[35] Vallochi AL, Nakamura MV, Schlesinger D, Martins MC, Silveira C, et al (2002) Ocular toxoplasmosis: more than just what meets the eye. Scand J Immunol 55: 324–328. 11967112 10.1046/j.1365-3083.2002.01052.x

[36] Streilein JW, Bradley D, Sano Y, Sonoda Y (1996) Immunosuppressive properties of tissues obtained from eyes with experimentally manipulated corneas. Invest Ophthalmol Vis Sci 37: 413–424. 8603847

[37] Miceli MV, Liles MR, Newsome DA (1994) Evaluation of oxidative processes in human pigment epithelial cells associated with retinal outer segment phagocytosis. Exp Cell Res 214: 242–249. 8082727 10.1006/excr.1994.1254

[38] Faqhiri Z, Bazan NG (2010) PI3K/Akt and mTOR/p70S6K pathways mediate neuroprotectin D1-induced retinal pigment epithelial cell survival during oxidative stress-induced apoptosis. Exp Eye Res 90: 718–725. 20230819 10.1016/j.exer.2010.03.002 PMC2873108

[39] Lee H, Chung H, Arnouk H, Lamoke F, Hunt RC, et al (2010) Cleavage of the retinal pigment epithelium-specific protein RPE65 under oxidative stress. Int J Biol Macromol 47: 104–108. 20510285 10.1016/j.ijbiomac.2010.05.014 PMC5158184

[40] da Silva CV, da Silva EA, Cruz MC, Chavrier P, Mortara RA (2009) ARF6, PI3-kinase and host cell actin cytoskeleton in *Toxoplasma gondii* cell invasion. Biochem Biophys Res Commun 378: 656–661. 19061866 10.1016/j.bbrc.2008.11.108

[41] Folkes AJ, Ahmadi K, Alderton WK, Alix S, Baker SJ, et al (2008) The identification of 2-(1H-indazol-4-yl)-6-(4-methanesulfonyl-piperazin-1-ylmethyl)-4-morpholin-4-yl-thieno[3,2-d]pyrimidine (GDC-0941) as a potent, selective, orally bioavailable inhibitor of class I PI3 kinase for the treatment of cancer. J Med Chem 51: 5522–5532. 18754654 10.1021/jm800295d

[42] Kong D, Yamori T (2007) ZSTK474 is an ATP-competitive inhibitor of class I phosphatidylinositol 3 kinase isoforms. Cancer Sci 98: 1638–1642. 17711503 10.1111/j.1349-7006.2007.00580.x PMC11158993

[43] Wang Y, Weiss LM, Orlofsky A (2010) Coordinate control of host centrosome position, organelle distribution, and migratory response by *Toxoplasma gondii* via host mTORC2. J Biol Chem 285: 15611–15618. 20236941 10.1074/jbc.M109.095778 PMC2865287

[44] Shrestha SP, Tomita T, Weiss LM, Orlofsky A (2006) Proliferation of *Toxoplasma gondii* in inflammatory macrophages *in vivo* is associated with diminished oxygen radical production in the host cell. Int J Parasitol 36: 433–441. 16516217 10.1016/j.ijpara.2006.01.006 PMC3109651

[45] Fang FC (2004) Antimicrobial reactive oxygen and nitrogen species: concepts and controversies. Nat Rev Microbiol 2: 820–832. 15378046 10.1038/nrmicro1004

[46] Allen LA, Beecher BR, Lynch JT, Rohner OV, Wittine LM (2005) Helicobacter pylori disrupts NADPH oxidase targeting in human neutrophils to induce extracellular superoxide release. J Immunol 174: 3658–3667. 15749904 10.4049/jimmunol.174.6.3658

[47] Garcia-Garcia JC, Rennoll-Bankert KE, Pelly S, Milstone AM, Dumler JS (2009) Silencing of host cell CYBB gene expression by the nuclear effector AnkA of the intracellular pathogen Anaplasma phagocytophilum. Infect Immun 77: 2385–2391. 19307214 10.1128/IAI.00023-09 PMC2687357

[48] Lin M, Rikihisa Y (2007) Degradation of p22phox and inhibition of superoxide generation by Ehrlichia chaffeensis in human monocytes. Cell Microbiol 9: 861–868. 17087735 10.1111/j.1462-5822.2006.00835.x

[49] Lambeth JD (2004) NOX enzymes and the biology of reactive oxygen. Nat Rev Immunol 4: 181–189. 15039755 10.1038/nri1312

