# Supplementary figures and images for: Toxoplasma gondii Proliferation Require Down-Regulation of Host Nox4 Expression via Activation of PI3 Kinase/Akt Signaling Pathway
Source: PLoS One. 2013 Jun 18;8(6):e66306. doi: 10.1371/journal.pone.0066306 (PMC3688893; doi:10.1371/journal.pone.0066306)

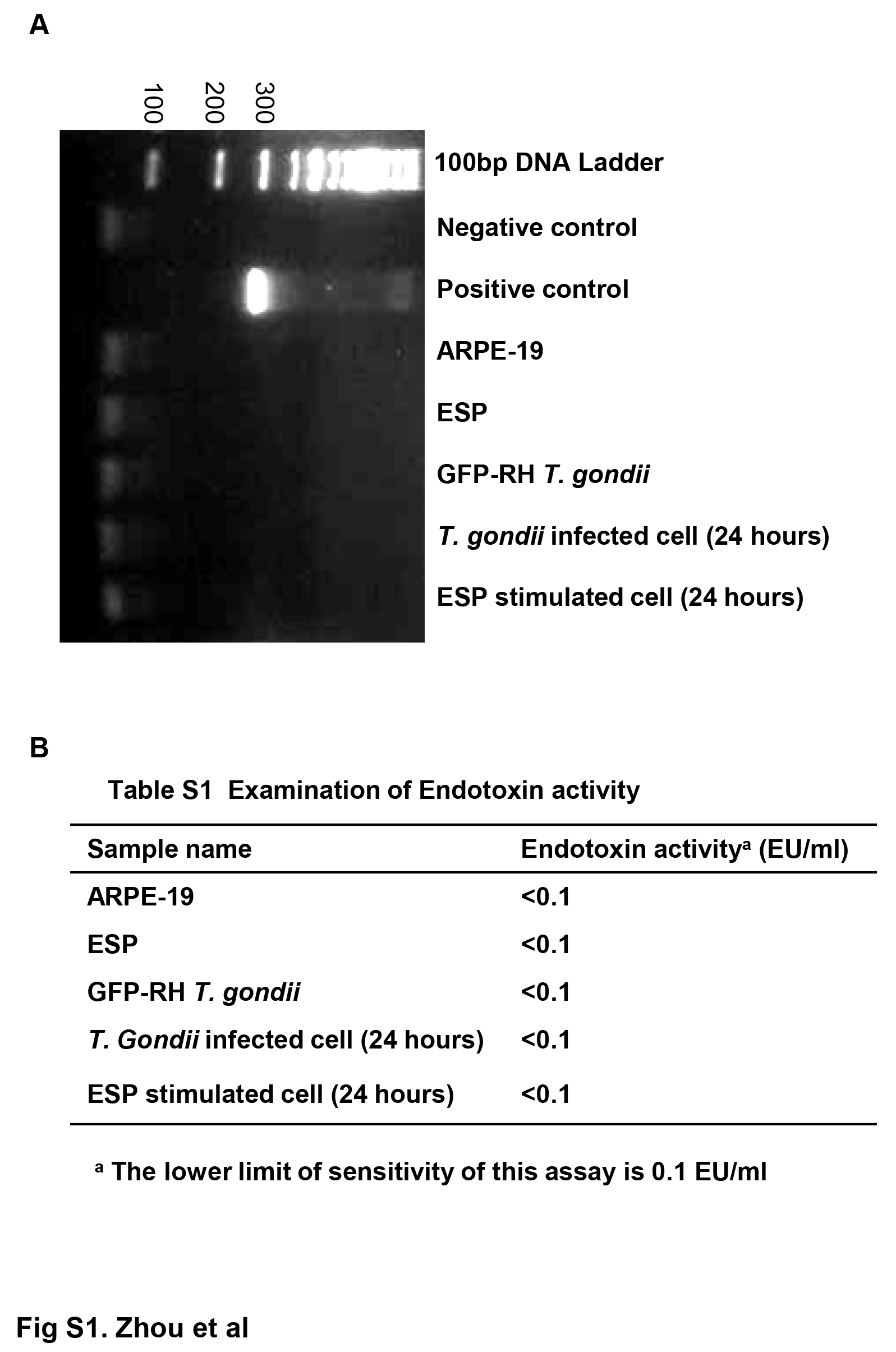

Supplement: Figure S1 — Examination of Mycoplasma and Endotoxin. (A) PCR analysis of ARPE-19 cell and parasite for detecting the presence of Mycoplasma DNA. The size of the PCR product obtained using the positive template with primer pairs is 270 bp. (B) Endotoxin levels of ARPE-19 cell and parasite detected with the LAL assay. (TIF) [file pone.0066306.s001.tif]

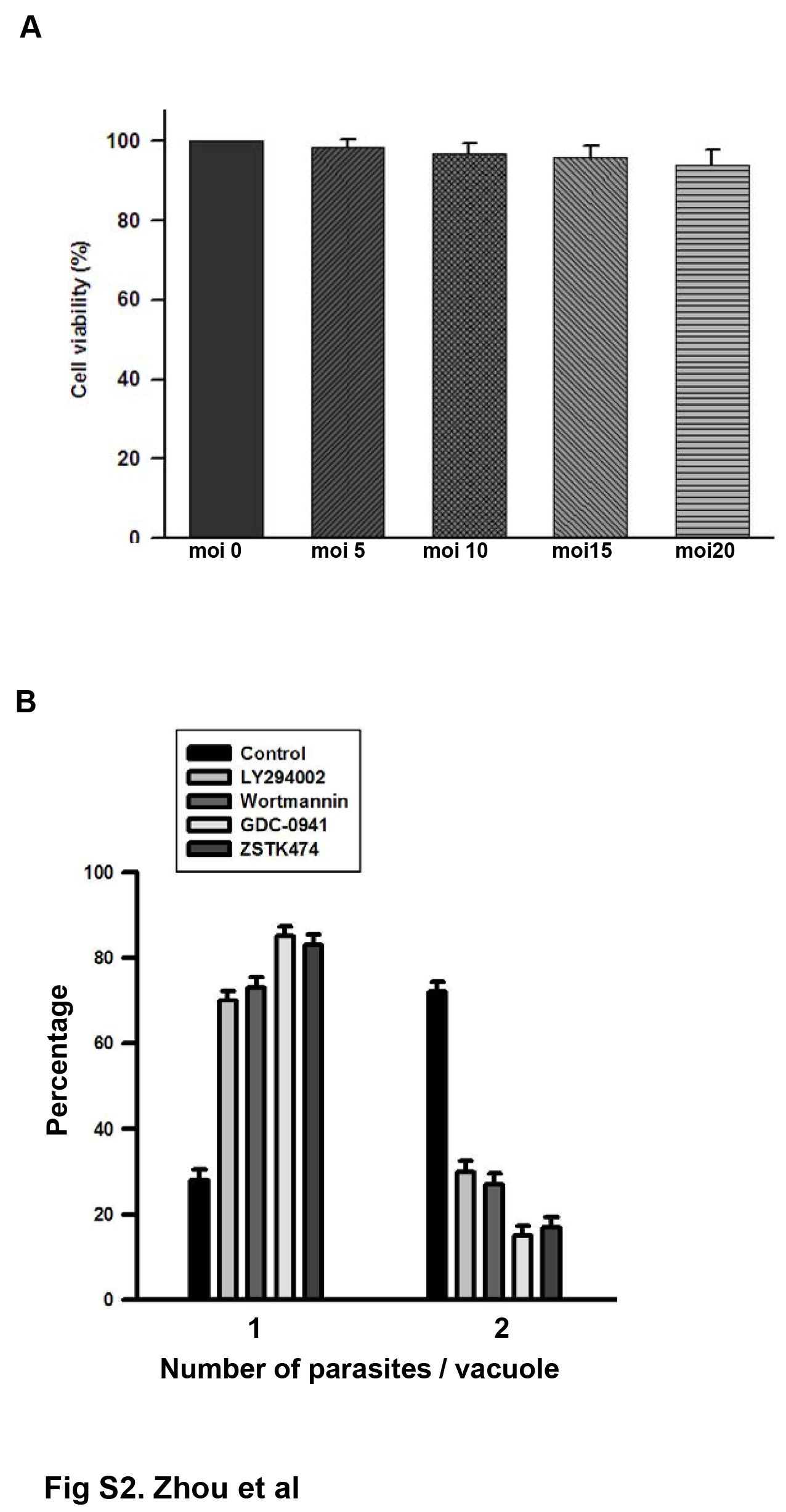

Supplement: Figure S2 — Confirmation of host viability after infection of T. gondii and effects of PI3K inhibitors on T. gondii replication ratio in 12 h. (A) ARPE-19 cells were infected with T. gondii at various moi for 24 h, and cell viability was assessed by MTT assay. (B) Cells were pre-incubated with PI3K inhibitors, either 10 µM LY294002, 200 nM wortmannin, or 250 nM GDC-0941, 10 nM ZSTK474 for 1 h. After washing, the cells were then infected with T. gondii at moi of 5 for 12 h. The number of parasites per vacuole was counted and converted in to percentage (doubling time of around 10 ∼12 h). (TIF) [file pone.0066306.s002.tif]

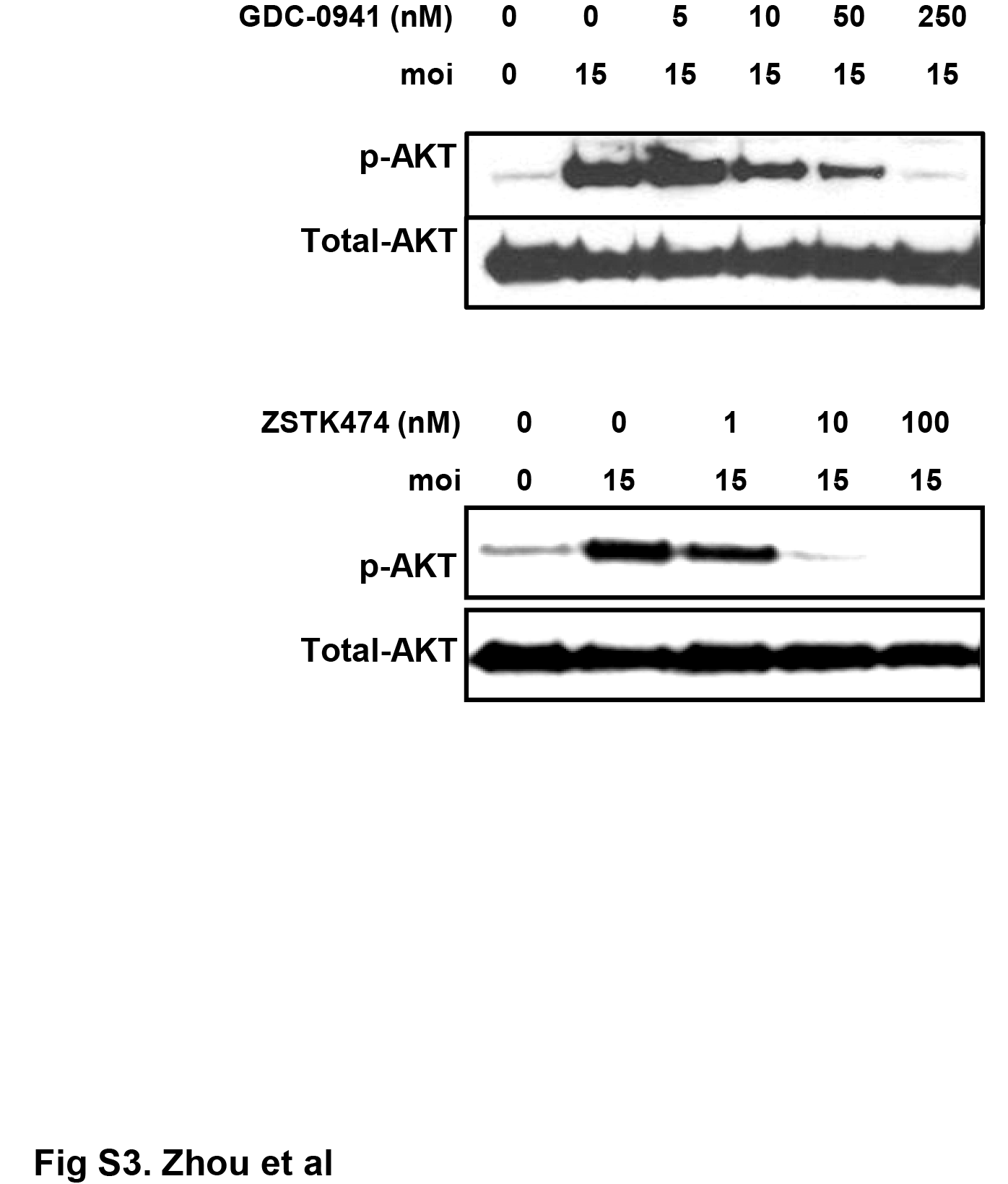

Supplement: Figure S3 — Effects of PI3K-specific inhibitors on Toxoplasma -induced PI3K/Akt activation. ARPE-19 cells were infected with T. gondii (moi 15) for 24 h and treated with increasing concentrations of GDC-0941 or ZSTK474 for the final 1 h. (TIF) [file pone.0066306.s003.tif]

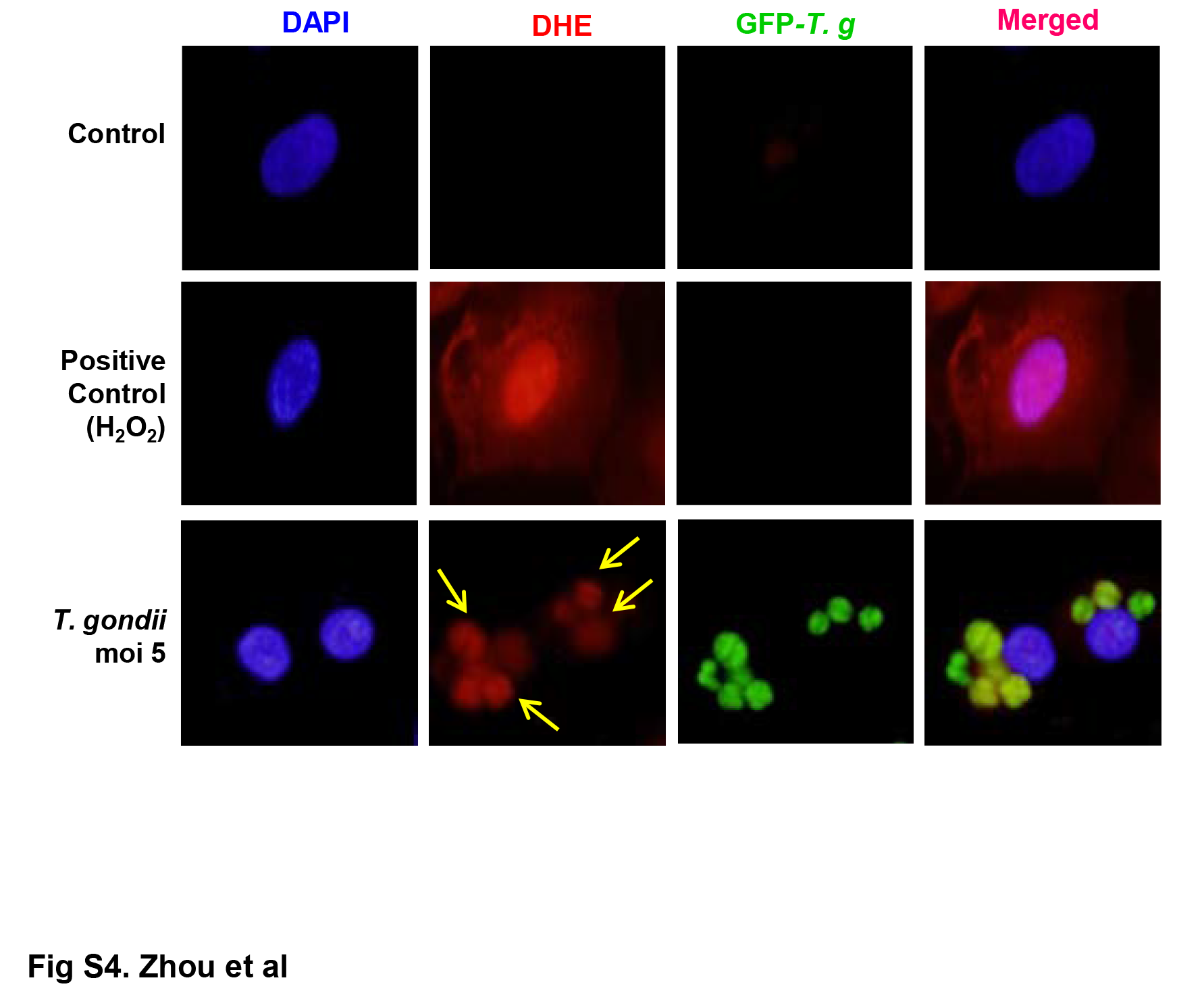

Supplement: Figure S4 — Presence of high ROS in T. gondii. Toxoplasma-induced ROS synthesis inhibition is suppressed by treatment of PI3K inhibitor. The arrows show that the T. gondii can be highly labeled by dihydroethidium (DHE). (TIF) [file pone.0066306.s004.tif]

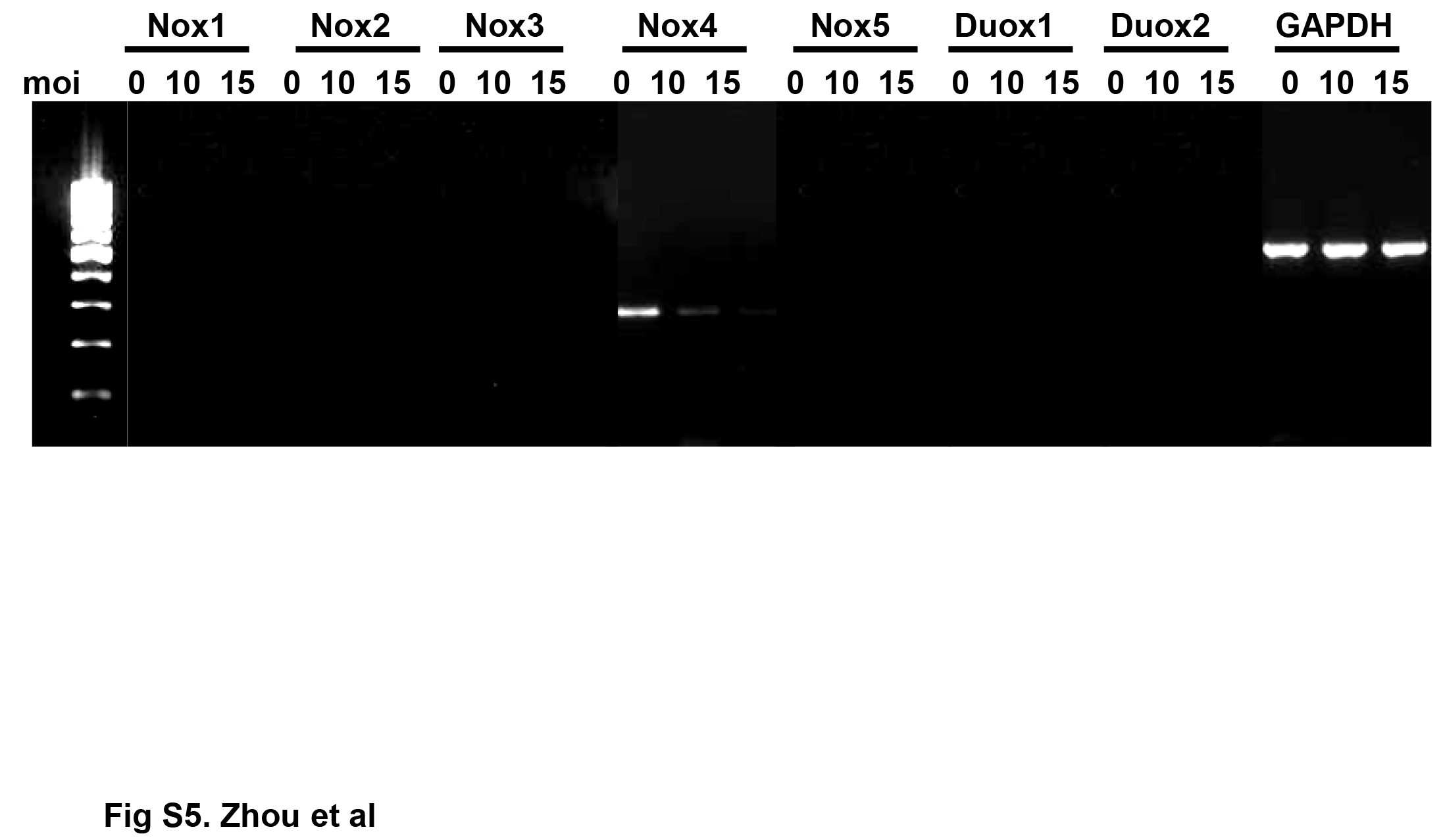

Supplement: Figure S5 — Messenger RNA expression pattern of NADPH oxidases in T. gondii infected ARPE-19 cell. Reverse transcription-PCR on whole RNA extracted from host cell with primer selected for seven catalytic subunits of NADPH oxidase isoforms (Nox1∼5, Duox1/Duox2). (TIF) [file pone.0066306.s005.tif]
